# I-TASSER server for protein 3D structure prediction

**DOI:** 10.1186/1471-2105-9-40

**Published:** 2008-01-23

**Authors:** Yang Zhang

**Affiliations:** 1 Center for Bioinformatics and Department of Molecular Bioscience, University of Kansas, 2030 Becker Dr, Lawrence, KS 66047, USA

## Abstract

**Background:**

Prediction of 3-dimensional protein structures from amino acid sequences represents one of the most important problems in computational structural biology. The community-wide Critical Assessment of Structure Prediction (CASP) experiments have been designed to obtain an objective assessment of the state-of-the-art of the field, where I-TASSER was ranked as the best method in the server section of the recent 7th CASP experiment. Our laboratory has since then received numerous requests about the public availability of the I-TASSER algorithm and the usage of the I-TASSER predictions.

**Results:**

An on-line version of I-TASSER is developed at the KU Center for Bioinformatics which has generated protein structure predictions for thousands of modeling requests from more than 35 countries. A scoring function (C-score) based on the relative clustering structural density and the consensus significance score of multiple threading templates is introduced to estimate the accuracy of the I-TASSER predictions. A large-scale benchmark test demonstrates a strong correlation between the C-score and the TM-score (a structural similarity measurement with values in [0, 1]) of the first models with a correlation coefficient of 0.91. Using a C-score cutoff > -1.5 for the models of correct topology, both false positive and false negative rates are below 0.1. Combining C-score and protein length, the accuracy of the I-TASSER models can be predicted with an average error of 0.08 for TM-score and 2 Å for RMSD.

**Conclusion:**

The I-TASSER server has been developed to generate automated full-length 3D protein structural predictions where the benchmarked scoring system helps users to obtain quantitative assessments of the I-TASSER models. The output of the I-TASSER server for each query includes up to five full-length models, the confidence score, the estimated TM-score and RMSD, and the standard deviation of the estimations. The I-TASSER server is freely available to the academic community at .

## Background

Protein structure prediction refers to the effort of generating 3-dimensional models from amino acid sequences using computer algorithms. However, structure modeling processes often involve human interventions because the human-expert knowledge combined with biochemical information (function, mutagenesis, catalytic residues, etc.) could help in both structural assembly and model selection [1,2]. Nevertheless, the development of fully-automated algorithms has the advantage in the potential application on proteome-scale structure predictions [3,4]. Especially, it allows non-experts to generate structural models for their own sequences through Internet services. In the recent community-wide blind experiment, CASP7, I-TASSER (as 'Zhang-Server') generated the best 3D structure predictions among all automated servers. The average GDT_TS [5] or TM-score [6] of all 124 targets/domains is at least 5% higher than the second best server and comparable with the best human-expert predictions [7].

Since the first public release in November 2006, the I-TASSER server has generated structure predictions for thousands of modeling requests from various laboratories in the world. We have been frequently asked by the users about how the quality of the I-TASSER models should be annotated because this will essentially decide how they will exploit the predictions in their research. The general idea of the modeling quality estimation of 3D models has been pursued by a number of authors [8-10], which merges as a new research topic of "model quality assessment programs" (MQAP) [11] and is assessed in the recent CASP7 experiment in the category of QA [12].

In this work, we introduce the on-line setting of the I-TASSER server and develop a confidence scoring system which can provide the users with a simple and reliable assessment of the I-TASSER models. Different from most of the MQAP programs that assess models purely based on the structure of the final models, the confidence scoring function developed here incorporates the information and parameters of the modeling simulations.

## Implementation

### I-TASSER method

I-TASSER is a hierarchical protein structure modeling approach based on the secondary-structure enhanced Profile-Profile threading Alignment (PPA) [13] and the iterative implementation of the Threading ASSEmbly Refinement (TASSER) program [14]. The detail of the I-TASSER method has been described in [15,16]. Here we give a brief overview of the method.

The target sequences are first threaded through a representative PDB structure library (with a pair-wise sequence identity cut-off of 70%) to search for the possible folds by four simple variants of PPA methods, with different combinations of the hidden Markov model [17] and PSI-BLAST [18] profiles and the Needleman-Wunsch [19] and Smith-Waterman [20] alignment algorithms. The continuous fragments are then excised from the threading aligned regions which are used to reassemble full-length models while the threading unaligned regions (mainly loops) are built by *ab initio *modeling [21]. The conformational space is searched by replica-exchange Monte Carlo simulations [22]. The structure trajectories are clustered by SPICKER [23,24] and the cluster centroids are obtained by the averaging the coordinates of all clustered structures. To rule out the steric clashes on the centroid structures and to refine the models further, we implement the fragment assembly simulation again, which starts from the cluster centroid of the first round simulation. Spatial restraints are extracted from the centroids and the PDB structures searched by the structure alignment program TM-align [25], which are used to guide the second round simulation. Finally, the structure decoys are clustered and the lowest energy structure in each cluster is selected, which has the C_α _atoms and the side-chain centers of mass specified. Pulchra [26] is used to add backbone atoms (N, C, O) and Scwrl_3.0 [27] to build side-chain rotamers.

If any region with >80 residues has no aligned residues in at least two strong PPA alignments of Z-score > Z_0 _(see below), the target will be judged as a multiple domain protein and domain boundaries are automatically assigned based on the borders of the large gaps. I-TASSER simulations will be run for the full chain as well as the separate domains. The final full-length models are generated by docking the model of domains together. The domain docking is performed by a quick Metropolis Monte Carlo simulation where the energy is defined as the RMSD of domain models to the full-chain model plus the reciprocal of the number of steric clashes between domains. The goal of the docking is to find the domain orientation that is closest to the I-TASSER full-chain model but has the minimum steric clashes. This procedure does not influence the multiple domain proteins which have all domains completely aligned by the PPAs.

### C-score

The C-score of the I-TASSER models is defined as

(1) $$\text{C-score}=\text{ln}\left(\frac{\text{M}}{{\text{M}}_{\text{tot}}}\cdot \frac{\text{1}}{\langle \text{RMSD}\rangle }\cdot \frac{\prod_{i=1}^{4}\text{Z}(i)}{\prod_{i=1}^{4}{\text{Z}}_{\text{0}}(i)}\right)$$

where M is the multiplicity of structures in the SPICKER cluster; M_tot _is the total number of the I-TASSER structure decoys used in the clustering; ⟨RMSD⟩ is the average RMSD of the decoys to the cluster centroid; Z(*i*) is the highest Z-score (the energy to mean in the unit of standard deviation) of the templates by the *i*th PPA threading program and Z_0_(*i*) is a program-specified Z-score cutoff for distinguishing between good and bad templates, i.e. Z_0_(1) = 7.0, Z_0_(2) = 8.5, Z_0_(3) = 8.0, Z_0_(4) = 10.5.

The first two factors of Equation 1 account for the degree of structure convergence in the SPICKER clustering, which correlates with the consistency of the external restraints and the inherent I-TASSER potential. The third factor accounts for the quality of threading alignments. The logarithm in Equation 1 is to adjust the C-score values in an approximately even distribution. A previously defined C-score has been shown to have a strong correlation with the quality of the predicted models [14]. Here, the definition of C-score is slightly different. First, a normalized Z-score by Z_0 _is used instead of the Z-score itself which makes it easy to extend the definition to the cases when templates are generated by different threading algorithms. Second, it accounts for the consensus of alignment confidence of multiple threading programs rather than one threading program.

We also tried other alternatives for the C-score definition. For example, if we add ⟨TM-score⟩, the average TM-score of the decoys to the cluster centroid, in the numerator of the second factor in Equation 1, the correlation between the C-score and TM-score will increase by ~2%. But it does not increase the correlation of C-score with RMSD and the calculation of ⟨TM-score⟩ will increase the SPICKER running time by ~20%. So we did not include ⟨TM-score⟩ in the C-score definition. We also attempted to optimize the powers of the three factors of Equation 1 by maximizing the correlation between C-score and the quality of final models in the training proteins. Interestingly, the optimized powers of all three factors are close to 1, which indicates that the C-score in Equation 1 is close to an optimal definition if considering these 3 factors.

### TM-score

TM-score is defined to assess the topological similarity of two protein structures [6]:

(2) $$\text{TM-score}=\frac{1}{L}\sum_{i=1}^{L}\frac{1}{1+d_{i}^{2}/d_{0}^{2}},$$

where *d*_*i *_is the distance of the *i*th pair of residues between two structures after an optimal superposition, $d_{0}=1.24\sqrt[{3}]{L-15}-1.8$, and *L *is the protein length. TM-score stays in [0, 1] with higher values indicating better models. Statistically, a TM-score ≤ 0.17 corresponds to a similarity between two randomly selected structures from the PDB library; a TM-score > 0.5 corresponds approximately to two structures of the similar topology. One advantage of the TM-score is that the meaning of the TM-score cutoffs is independent of the size of proteins [6].

### Server setting

The URL address of the on-line I-TASSER server is listed at the end of the paper. To use the server, what users need to provide is the amino acid sequence of the proteins to be modeled in the FASTA format. Currently, the acceptable size range of the targets is between 10–1,500 residues. Depending on the protein size, the I-TASSER modeling procedure takes a maximum of 48 hours (typically 5–10 hours for a sequence around 200 residues). After the modeling is finished, an email will be sent to the users, which include the PDB format files of up to 5 predicted models, C-score of the models, and the predicted RMSD and TM-score of the first model. A brief explanation of the RMSD, TM-score, and C-score is also provided in the email.

Once a prediction is made, a GIF visual file is made for each of the I-TASSER models so that the users can get a quick on-line view of how the topology of their models looks like. The PDB files and the visual files are kept on our server for 365 days and made publicly downloadable at [28], so that other users can quickly retrieve the modeling results without resubmitting the jobs when they want to model the same or similar proteins. The queue of the jobs is shown on the page as well so that the users can track their submitted jobs. Finally, an "About I-TASSER server" webpage [29] is designed to provide a detailed introduction of the server which is kept updated when new features are developed.

## Results and Discussions

For the benchmark of the I-TASSER server, we collect 800 nonhomologous single-domain proteins directly from the PDB library [30], which have a pair-wise sequence identity <30% with the size ranging from 50 to 300 residues. The purpose has been made to have the selected proteins of a balanced distribution in secondary structure classes and modeling difficulty. As a result, the benchmark set includes 220/212/368 α/β/αβ-proteins. Based on the Z-scores of the PPA alignments, 236/248/316 targets are assigned as easy/medium/hard targets respectively. We randomly select 300 proteins as the training set to fit the parameters of the estimated model quality (see below); the remaining 500 proteins will be used as the test set (see [31]). When I-TASSER is used to generate models for the 800 proteins, homologous templates with sequence identity >30% to the target are excluded from the threading template library.

It should be mentioned that here we benchmark the I-TASSER algorithm only on the single-domain proteins. For multiple-domain proteins, a small misorientation of the domains may result in dramatic change in TM-score and RMSD values even if the topology of the individual domains is unchanged, which can result in divergent correlations of the C-score and the overall model qualities. Consequently, the confidence score and quality estimation of multiple-domain models should be understood approximately as those for the individual domain units.

### Correlation of C-score and model qualities

**Figure 1:**
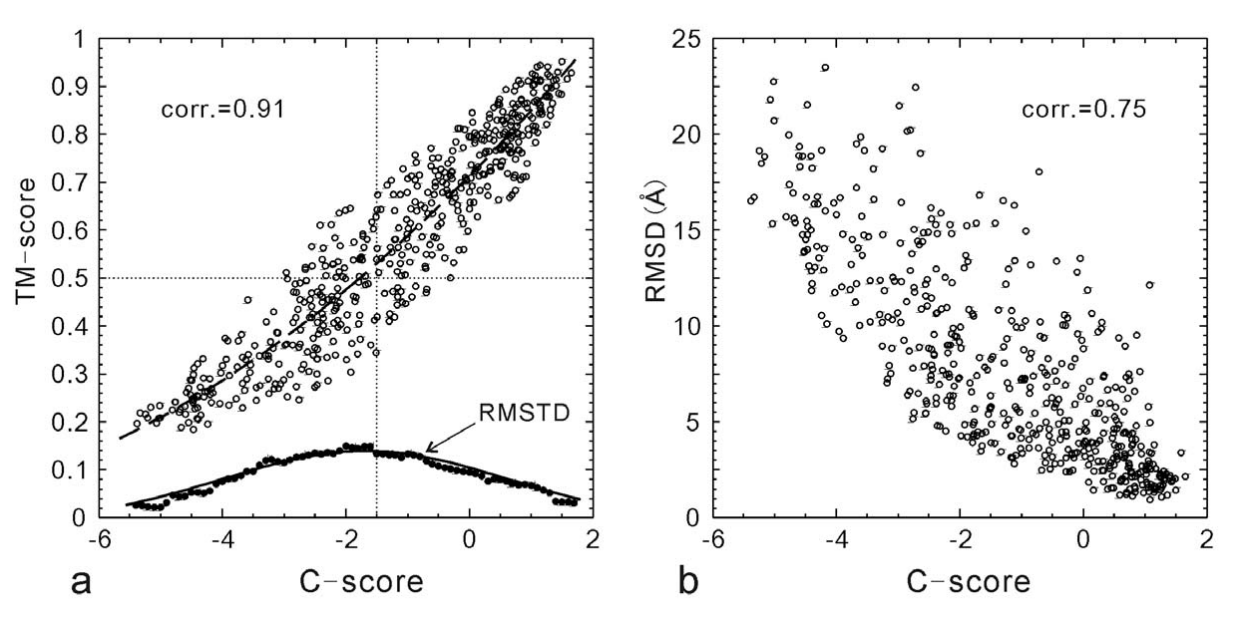
TM-score (a) and RMSD (b) versus C-score of the I-TASSER models for 500 testing proteins. The dashed curve in (a) is from Equation 3 which is fit from the 300 training proteins and used for estimating the TM-score of the I-TASSER models. The solid circles are the root mean squared deviation from the estimated TM-score values (RMSTD). The solid curve is from Equation 4 which is fit from the 300 training proteins. The dotted lines are the TM-score and C-score cutoffs for correct folds.

In Figure 1a, we display the TM-score of the first I-TASSER models of all 500 testing proteins, which shows a strong correlation to the C-scores with a Pearson correlation coefficient of 0.91. If we define a model of TM-score > 0.5 as a correct fold and assess the models using a cutoff of C-score > -1.5, the false-positive and false-negative rate are 0.05 and 0.09 respectively.

**Figure 2:**
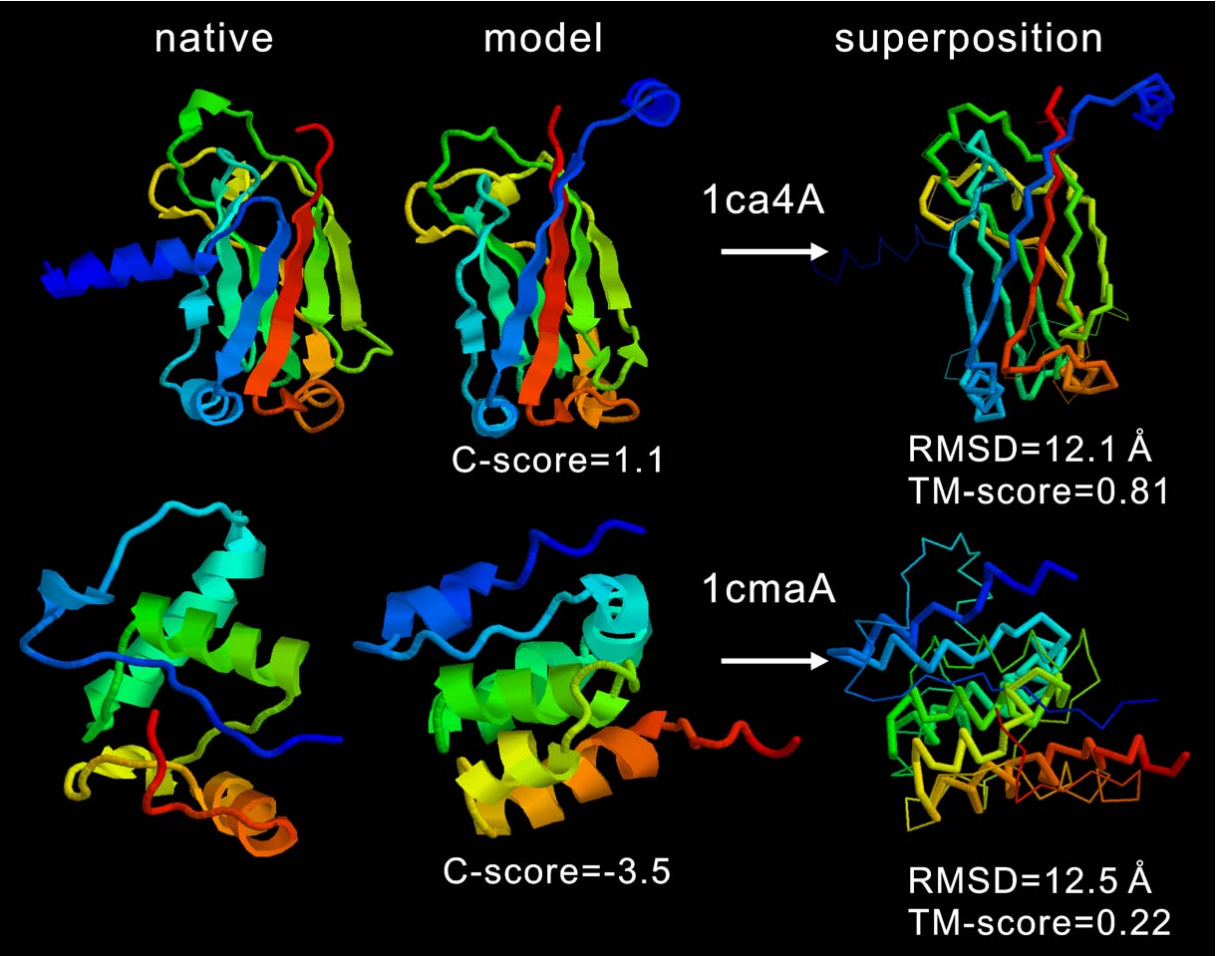
Two examples of the I-TASSER models from 1ca4A and 1cmaA. Both models have similar RMSD values but indicate significantly different modeling qualities. In the superposition, the thin backbones are the native structure and thick backbones the I-TASSER models. Blue to red runs from N- to C-terminal.

The correlation of RMSD with the C-score is not as strong as that of the TM-score (Figure 1b). Many high C-score models have a big RMSD. This is mainly because of the definition of RMSD which averages distances of all residue pairs with an equal weight [32]. Therefore, a big local modeling error will result in a high RMSD value even when the global topology is correct. For illustration, in Figure 2, we show two examples of the I-TASSER modeling. For 1ca4A which has a high C-score = 1.1, the core region of the model is very close to the native with a RMSD = 2.2 Å. But the N-terminus of the model is mis-orientated which results in an overall RMSD = 12.1 Å, a region usually implying wrong folds. As defined in Equation 2, TM-score weights the residue pairs of small distances stronger than those of large distances, which is not sensitive to the local structure errors and has a value of 0.81 in the example. For 1cmaA, the global topology of the secondary structure arrangements in the I-TASSER modeling is incorrect with a TM-score of 0.22 (close to random). The C-score in this case is -3.5. However, the RMSD (= 12.5 Å) is similar as that of 1ca4A. Therefore, the RMSD values in the high RMSD region are not sensitive to the global topology of structures.

**Figure 3:**
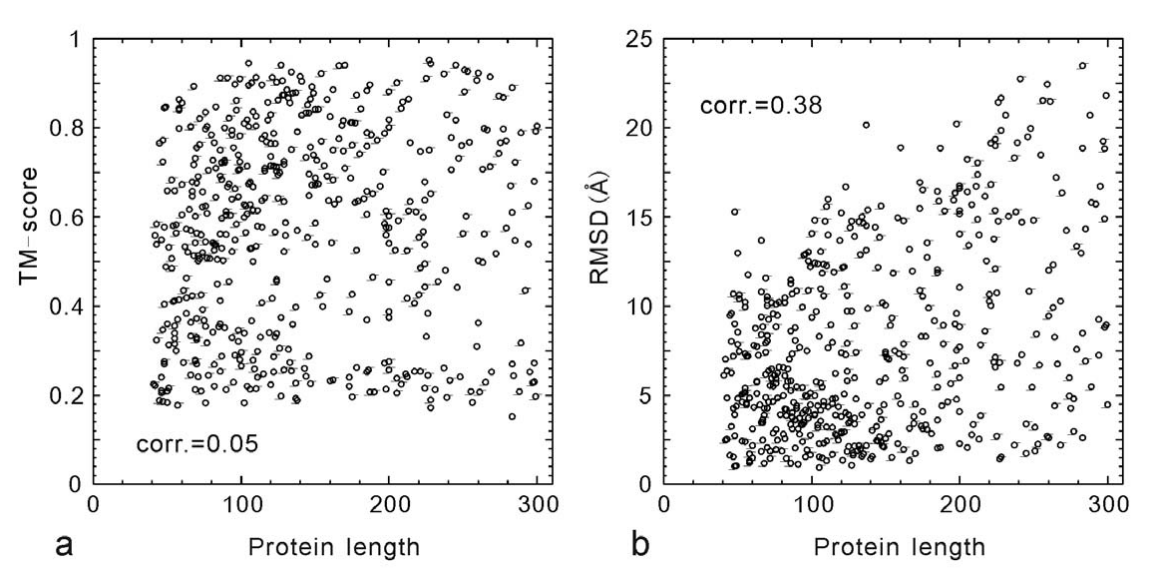
TM-score (a) and RMSD (b) of the I-TASSER models versus the length of target proteins. The numbers indicate the Pearson correlation coefficients.

The second reason for the low RMSD/C-score correlation is due to the inherent size dependence of RMSD. In Figure 3, we show the TM-score and RMSD values of the I-TASSER models versus the protein length for the 500 test proteins. Obviously, the small proteins tend to have a lower RMSD, a tendency also seen in the randomly selected PDB structure pairs [6,33], which results in a non-trivial RMSD/length correlation (Figure 3b). Since the distance in TM-score is normalized by a length dependent scale (see Equation 2), there is no length dependence in the TM-score values, which have an almost uniform cut near 0.17 (Figure 3a).

**Figure 4:**
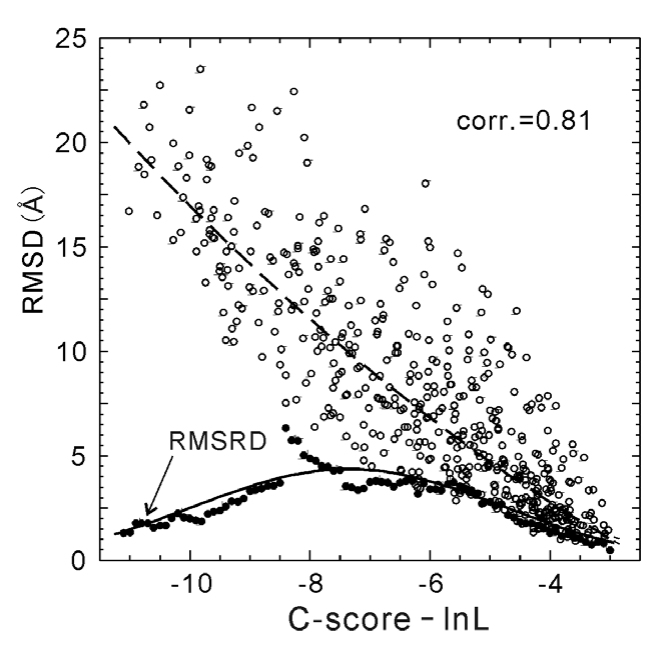
RMSD versus C-score-ln(*L*) of the I-TASSER models for 500 test proteins (open circles). The dashed curve is from Equation 5 which is fit from the 300 training proteins and used for estimating RMSD of the I-TASSER models. The solid circles are the root mean squared RMSD deviation (RMSRD) from the estimated RMSD values. The solid curve is from Equation 6 which is fit from the 300 training proteins.

In Figure 4, we plot the RMSD values versus C-score-ln(*L*), which has an obviously stronger correlation (correlation coefficient = 0.81) than that in Figure 1b.

As a control, we also calculate the correlation of TM-score (or RMSD) with the sequence identity between the target and the best template, which is 0.33 (or -0.23). The low correlation is not surprising because all homologous templates with a high sequence identity >30% have been excluded and the profile-profile programs often identify templates of correct topology even when the sequence identity to the target is low.

### Quantitative estimate of the quality of I-TASSER models

Based on the I-TASSER models of the 300 training proteins, we fit a two-order polynomial to the TM-score/C-score data by the least square fitting method [34]. We obtain

(3) Tm-score = 0.0006*C-score^2 ^+ 0.13*C-score + 0.71,

with a root mean squared TM-score deviation (RMSTD) of 0.08 for the training protein set. In Figure 1a, we show the curve of Equation 3 (dashed curve) which fits very well with the test proteins with a RMSTD of 0.09. If we consider Equation 3 as the estimated TM-score, the average error of the estimation is 0.08 in the test set. Here we note that the RMSTD is defined as sqrt⟨(TM-score - ⟨TM-score⟩)^2^⟩ and the average error of estimation is ⟨|TM-score - ⟨TM-score⟩|⟩, where ⟨TM-score⟩ is the average TM-score in the training set and the estimated TM-score in the test set. If we use RMSTD as the standard deviation of the TM-score estimation, there is a probability of 68.3% that the real TM-score will fall in the range of TM-score ± RMSTD [34].

In the lower part of Figure 1a, we show the data of RMSTD versus C-score. At each point, the RMSTD from the estimated TM-score by Equation 3 is calculated for the proteins in a bin of [C-score-0.5, C-score+0.5]. On average, each bin contains 70 proteins. The dependence of RMSTD with C-score is spindle-like, which indicates that the TM-score can be relatively easier predicted in both high and low C-score regions compared with that in the medium C-score region. The data fits well with the Gaussian function in the training proteins as

(4) $$\text{RMSTD}=0.14\exp\left(-\frac{{\left(\text{C-score}+1.7\right)}^{2}}{10.7}\right).$$

An overlap of Equation 4 with the RMSTD data is shown in Figure 1a (solid curve).

Since the RMSD of the I-TASSER models correlates better with C-score-ln(*L*) than with C-score, we fit the 2-order polynomial with the data of RMSD/C-score-ln(*L*) in the 300 training proteins. We obtain

(5) RMSD = 0.09(C-score - ln *L*)^2 ^- 1.14(C-score - ln *L*) - 3.17,

with a root mean squared RMSD deviation (RMSRD) of 3.1 Å. In Figure 4, we show the curve of Equation 5 (dashed curve) which fits well with the testing proteins with a RMSRD = 3.7 Å. The average error of the estimated RMSD using Equation 5 is 2.0 Å in the test set.

In the low part of Figure 4, we display the RMSRD value calculated in each bin of [C-score-ln*L*-0.5, C-score-ln*L*+0.5] and the Gaussian curve fitted from the training proteins, i.e.

(6) $$\text{RMSRD}=4.5\exp\left(-\frac{{\left(\text{C-score}-\ln L+7.4\right)}^{2}}{13.7}\right).$$

## Conclusion

We develop the I-TASSER server for the automated full-length protein structure prediction. A series of accessorial WebPages are designed to facilitate the users in submitting, viewing and tracking the predictions. Based on the statistical significance of the PPA threading alignments and the structure convergence of the Monte Carlo simulations, a new confidence score (C-score) is introduced and benchmarked for the I-TASSER server, which demonstrates a strong correlation with the real quality of the final models. The Pearson correlation coefficients of the C-score with TM-score and RMSD are 0.91 and 0.75 respectively. The strong correlation data allows us to make quantitative estimates of the accuracy of the I-TASSER predictions. Using a 2-order polynomial equation fit from 300 training proteins, we can predict the TM-score and RMSD of the final models with an average error of 0.08 and 2.0 Å respectively in a large scale benchmark test.

For each submitted sequence, following items will be returned to the users by email after the I-TASSER modeling: (1) up to five predicted models ranked based on the structure density of the SPICKER clustering; (2) C-score of all the I-TASSER models; (3) estimated TM-score and RMSD for the first model in the form of *Estimation *± *Deviation *where the values of *Estimation *and *Deviation *are calculated by Equations 3–6. By definition, in 68.3% of cases, the real TM-score and RMSD values will fall in this range [34]. Despite the significant correlation between the C-score and the TM-score, they have been introduced for the different purposes. While the C-score judges how confident the server feels about the predictions based on the information from the modeling simulations, TM-score is a measure of the absolute quality of the final model in comparison with the native structure, which is estimated through the calculation of the C-score.

It should be mentioned that the estimated qualities are provided only for the first model, although for the purpose of providing more information the C-score of all 5 models are sent to the users. The correlation of C-score and modeling quality for the lower-rank models is much weaker than that for the first model. This is understandable because the conformational space covered by the I-TASSER simulations is limited. For easy targets almost all decoys are near-native and the structures are mainly clustered in the first cluster. After removing the structures in the first cluster, the size of the lower-rank clusters will be much smaller which may be comparable to that of hard targets. But the quality of the lower-rank clusters from the easy targets is still on average better than that from the hard targets because most decoys generated in the hard targets are incorrect. Nevertheless, there is a correlation between the rank and the quality of the clusters for the same target. In this set of test proteins, the average TM-score (RMSD) of the top-five models are 0.501 (9.6 Å), 0.468 (10.6 Å), 0.466 (10.7 Å), 0.461 (11.1 Å), and 0.454 (11.3 Å) respectively. Therefore, the C-score and predicted data should be considered as an upper-limit estimate for the quality of all I-TASSER models.

## Availability and requirements

Project name: I-TASSER server

Project home page: 

Operating system(s): Windows, Linux, Mac

Programming language: Perl, Fortran77

License: GPL

Any restrictions to use by non-academics: license needed

## Abbreviations

I-TASSER: iterative threading assembly refinement algorithm.

PPA: profile-profile alignment threading algorithm.

RMSD: root mean squared deviation.

RMSRD: root mean squared RMSD deviation from average or estimated RMSD.

RMSTD: root mean squared TM-score deviation from average or estimated TM-score.

## Authors' contributions

YZ developed the I-TASSER server, performed the benchmark calculation and wrote the manuscript. He has read and approved the final manuscript.
